# Chain Substituted Cannabilactones with Selectivity for the CB2 Cannabinoid Receptor

**DOI:** 10.3390/molecules24193559

**Published:** 2019-10-01

**Authors:** Shakiru O. Alapafuja, Spyros P. Nikas, Thanh C. Ho, Fei Tong, Othman Benchama, Alexandros Makriyannis

**Affiliations:** 1 Center for Drug Discovery and Department of Pharmaceutical Sciences, Northeastern University, Boston, MA 02115, USA; s.alapafuja@northeastern.edu (S.O.A.); t.ho@northeastern.edu (T.C.H.); tong.f@husky.neu.edu (F.T.); othmanb@gmail.com (O.B.); 2 Department of Chemistry & Chemical Biology, Northeastern University, Boston, MA 02115, USA

**Keywords:** cannabinoid receptors, CB2 selective ligands, synthesis, cannabilactones, structure-activity relationship studies

## Abstract

In earlier work, we reported a novel class of CB2 selective ligands namely cannabilactones. These compounds carry a dimethylheptyl substituent at C3, which is typical for synthetic cannabinoids. In the current study with the focus on the pharmacophoric side chain at C3 we explored the effect of replacing the C1′-*gem*-dimethyl group with the bulkier cyclopentyl ring, and, we also probed the chain’s length and terminal carbon substitution with bromo or cyano groups. One of the analogs synthesized namely 6-[1-(1,9-dihydroxy-6-oxo-6*H*-benzo[*c*]chromen-3-yl) cyclopentyl] hexanenitrile (AM4346) has very high affinity (*K*_i_ = 4.9 nM) for the mouse CB2 receptor (mCB2) and 131-fold selectivity for that target over the rat CB1 (rCB1). The species difference in the affinities of AM4346 between the mouse (m) and the human (h) CB2 receptors is reduced when compared to our first-generation cannabilactones. In the cyclase assay, our lead compound was found to be a highly potent and efficacious hCB2 receptor agonist (EC_50_ = 3.7 ± 1.5 nM, *E_(max)_* = 89%). We have also extended our structure-activity relationship (SAR) studies to include biphenyl synthetic intermediates that mimic the structure of the phytocannabinoid cannabinodiol.

## 1. Introduction

The two G-protein coupled receptors (GPCRs) termed cannabinoid receptor 1 (CB1) and cannabinoid receptor 2 (CB2) are principal components of the endocannabinoid biosignaling system and molecular targets for the psychoactive constituent of cannabis Δ^9^-tetrahydrocannabinol (Δ^9^-THC) [1,2,3]. Both CB1 and CB2 also bind the FDA approved drug Nabilone and many other exogenous (plant-derived and synthetic) cannabinoids. The CB1 receptor retains a high (≥97%) degree of amino acid sequence identity across mouse, rat, and human [4]. In contrast, the human CB2 receptor displays only 82% and 81% amino acid identity with mouse [5] and rat [6] respectively. The CB1 receptor was found to be localized primarily in the brain and it is one of the most abundant GPCRs in the central nervous system (CNS) [4]. This receptor is also found in peripheral tissues and organs [7]. Activation of the central CB1 receptors mediates most of the cannabinoid psychotropic and behavioral effects. Conversely, the CB2 receptors are detectable at very low levels in brain and they are expressed predominantly in immune cells and in the periphery [8]. Nevertheless, the CB2 receptors may be induced in the CNS under pathologic conditions. Collaborative efforts, including our laboratory, have led to the first crystal structures of the agonist and antagonist bound human CB1, while recently we published the first crystal structure of the human CB2 receptor in a complex with the antagonist AM10257. This breakthrough work provides a molecular basis for predicting the binding modes of cannabinoids with their target proteins, and offers invaluable information for target-based drug design [9,10,11,12].

Selective CB2 receptor activation is a very promising method of modulating the endocannabinoid system because the pharmacologic effects are devoid of centrally mediated liabilities that are correlated with CB1 receptor modulation either directly by exogenous cannabinoids or indirectly through functional interactions with other receptor systems [13]. Selective CB2 activation would decrease CB1 associated ataxia, hypothermia, mood and memory disturbances, and abuse potential [14,15,16,17,18,19]. Currently, the CB2 receptor has emerged as an attractive therapeutic target for the treatment of inflammatory and neuropathic pain, as well as neurodegenerative disorders [20,21,22,23], and cancer [24,25,26].

**Figure 1:**
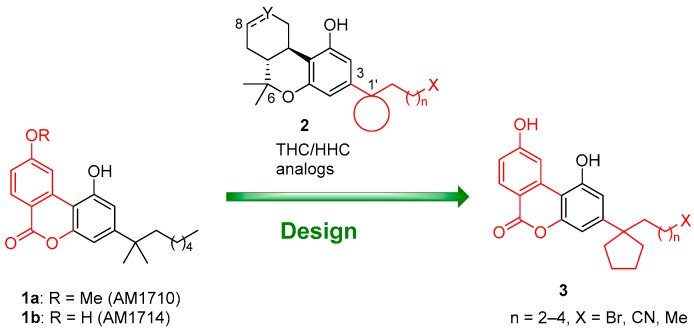
Design of chain substituted cannabilactones and structures of the first-generation analogs AM1710 and AM1714.

**Figure 2:**
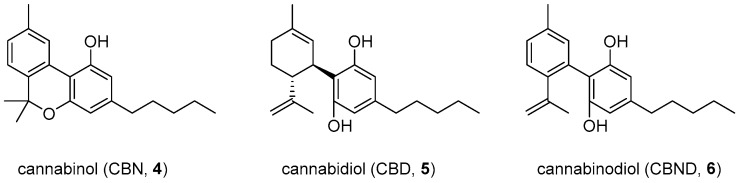
Chemical structures of the phytocannabinoids cannabinol (CBN), cannabidiol (CBD), and cannabinodiol (CBND).

Our group has identified the cannabilactones (Figure 1), as a new class of CB2 agonists with structural similarities to the 6,6-dimethyl counterpart and phytocannabinoid cannabinol (Figure 2). Two first generation analogs within this class of compounds namely AM1714 and AM1710 (Figure 1) have become important pharmacological tools in establishing proof of concept for the usefulness of the CB2 receptor activation approach [27,28,29,30,31,32,33]. Both these CB2 selective agonists are found to possess potent peripheral analgesic activity in several animal models of inflammatory and neuropathic pain. Moreover, functional studies with a lead compound have highlighted the potential of the cannabilactone based CB2 agonists to behave as neutral antagonists/low potency inverse agonists at CB1, a unique property which imparts very high functional selectivity for CB2 over CB1 [30,31,33]. Thus, from a medicinal chemistry perspective, the class of cannabilactone compounds remains an attractive structural motif for further development of CB2 selective and efficacious agonists.

The present work is informed by our findings with analogs of (−)-Δ^8^-tetrahydrocannabinol (THC) and (−)-hexahydrocannabinol (HHC) with cyclic moieties at the C1′ position of the side chain pharmacophore [34,35,36,37] (Figure 1). These studies have shown that analogs with six- to eight-atoms-long side chains substituted at C1′ with a cyclopentyl ring exhibit remarkably high affinities for CB1 and CB2 receptors. Taken together, in this study our design replaces the 1′,1′-*gem*-dimethyl group in the cannabilactone scaffold with the larger and sterically more confined cyclopentyl group (Figure 1). Additionally, we have explored the pharmacophoric limits of side chain length, while the polar characteristics have been enhanced by incorporating bromo- and cyano-substituents at the terminal carbon atom [37,38]. All synthesized compounds were assessed for their binding affinities at rat CB1 (rCB1) and mouse CB2 (mCB2) receptors while the most promising analogs were also tested in human CB2 (hCB2) to identify potential species differences between the mouse and human clone. One of the analogs synthesized namely AM4346 has very high affinity (*K*_i_ = 4.9 nM) and 131-fold selectivity for mCB2 over rCB1 and behaves as a potent CB2 agonist in the cyclase assay. The species difference in the affinities of AM4346 between the mouse and the human CB2 receptors is reduced when compared to our first-generation compound AM1714, while AM4346 is endowed with enhanced polarity due to the presence of the cyano group. Additionally, we extended our SAR to include the biphenyl synthetic intermediates **23a**–**23d** as they encompasses the biaryl subunit which is a privileged structure (biaryls found in 4.3% of known drugs), and also, they have structural similarities with the phytocannabinoid cannabidiol and its oxidative metabolite cannabinodiol (Figure 2) [39,40,41,42].

## 2. Results and Discussion

### 2.1. Chemistry

**Scheme 1:**
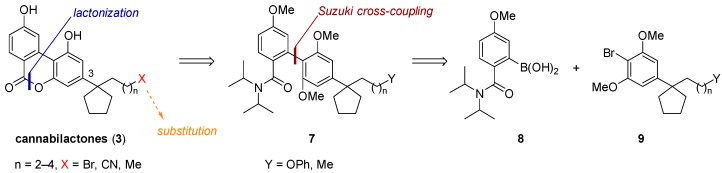
Retrosynthetic analysis of cannabilactones.

We conjectured that cannabilactones would be assessible from biphenyl compounds **7** via lactonization (Scheme 1). The bromo- or cyano-group on the C3-side chain of cannabilactones would be introduced to either the biphenyl or the cannabilactone template via substitution. Disconnection of biphenyl compounds **7** through a Suzuki coupling led to boronic acid **8** and aryl bromides **9**.

**Scheme 2:**
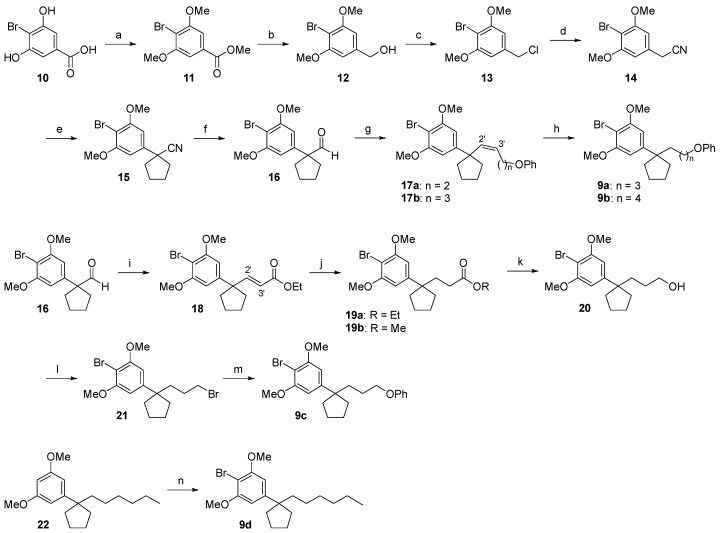
Synthesis of aryl bromides *^a^*.

The synthesis of aryl bromides **9a–9d** is summarized in Scheme 2. Bromides **9a–9c** were prepared in a general approach starting from commercially available 4-bromo-3,5-dihydroxybenzoic acid (**10**). Following the procedure of Luning et al. [43], etherification of the phenolic hydroxyl groups as well as, esterification of the carboxylic acid was accomplished in a single step by refluxing a mixture of **10**, potassium carbonate, and dimethyl sulfate in acetone. Reduction of the resulting ester **11** with diisobutylaluminum hydride (DIBAL-H) gave benzylic alcohol **12**. Although the conversion of **12** to the benzyl chloride has been reported [44], we adopted a more convenient and higher yielding approach. This involved refluxing a mixture of **12** and triphenylphosphine in dry carbon tetrachloride for two hours [35,45,46] to give **13** in 93% yield. Treatment of **13** with sodium cyanide in dimethyl sulfoxide afforded benzyl nitrile **14** in 87% yield [35,44]. Sequential deprotonation of **14** using potassium bis(trimethylsilyl)amide and cyclobisalkylation using 1,4-dibromobutane in tetrahydrofuran (THF) at 0 °C produced cyclopentyl nitrile **15** in 70% yield [35,47]. This was transformed to aldehyde **16** in 85% yield with DIBAL-H at −78 °C [48].

*^a^* Reagents and conditions: (a) Me_2_SO_4_, K_2_CO_3_, acetone, reflux, 92%; (b) DIBAL-H, THF, rt, 1 h, 85%; (c) Ph_3_P, CCl_4_, reflux, 93%; (d) NaCN, DMSO, rt, overnight, 87%; (e) KHMDS, 1,4-dibromobutane, THF, 0 °C, 5 min, 70%; (f) DIBAL-H, CH_2_Cl_2_, −78 °C, 1 h, 85%; (g) (3-phenoxypropyl)- or (4-phenoxybutyl)triphenylphosphonium bromide, KHMDS, THF, 0 °C, 10 min, 94% for **17a** and 93% for **17b**; (h) H_2_, Pd/C, EtOAc, rt, 12 h, 95% for **9a** and 94% for **9b**; (i) triethyl phosphonoacetate, NaH, THF, 92%; (j) for **19a**: H_2_, Pd/C, EtOAc, 30 psi, 95%; for **19b**: Mg/MeOH, 0 °C, 2 h, then rt, 12 h, 80%; (k) DIBAL-H, THF, rt, 1 h, 86% from **19a**, 85% from **19b**; (l) Ph_3_P/CBr_4_, 0 °C → rt, 85%; (m) NaOPh, DMSO, rt, 20 h, 50%; (n) Br_2_, CCl_4_, 0 °C, 1 h, 78%.

Wittig olefination of aldehyde **16** with (3-phenoxypropyl)- and (4-phenoxybutyl) triphenylphosphonium bromides gave exclusively *cis*-alkenes **17a** and **17b**, respectively, in 93–94% yields. The geometry of the newly formed double bond in **17a** and **17b** was assigned based on the ^1^H NMR spectra of crude products (^3^*J*_H2′−H3′_ ~ 11.3 Hz). Reduction of these alkenes by catalytic hydrogenation afforded the corresponding aryl bromides **9a** and **9b**. Wittig olefination of aldehyde **16** with (2-phenoxyethyl)triphenylphosphonium bromide was not successful because vinyltriphenylphosphonium bromide was generated under the reaction conditions [49]. To prepare aryl bromide **9c**, a slightly modified route was followed. Horner-Wardsworth-Emmons olefination of aldehyde **16** with commercially available triethyl phosphonoacetate [50,51,52] produced exclusively *trans*-conjugated ester **18** in 92% yield (^3^*J*_H2′−H3′_ ~ 15.8 Hz). Palladium-catalyzed hydrogenation of **18** in ethyl acetate required high pressure (30 psi) to produce ethyl ester **19a** in 95% yield, whereas exposure of **18** to magnesium turnings in methanol gave methyl ester **19b** in 80% yield [53]. Reduction of either **19a** or **19b** with DIBAL-H at room temperature gave alcohol **20** (85–86% yield). This was followed by treatment of **20** with triphenylphosphine and carbon tetrabromide to afford **21** in 85% yield. Phenoxide displacement of **21** in dimethyl sulfoxide provided aryl bromide **9c** in moderate yield (50%). Additionally, aryl bromide **9d** was prepared via bromination of resorcinol dimethyl ether **22** [35] using bromine in carbon tetrachloride (78% yield) [54].

**Scheme 3:**
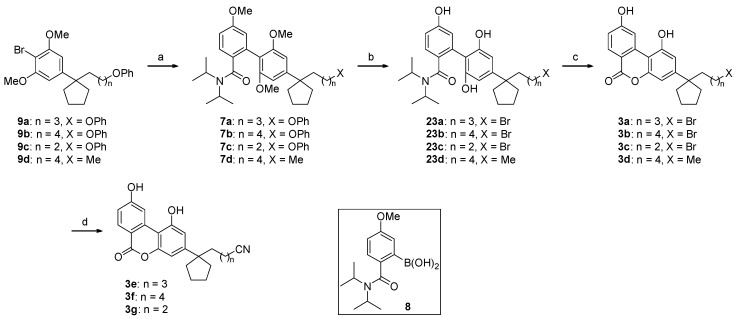
Synthesis of cannabilactones *^a^*.

With aryl bromides **9a**–**9d** in hand, we proceeded to complete the synthesis of cannabilactones (Scheme 3). Suzuki cross-coupling of aryl bromides **9a**–**9d** with boronic acid **8**, which was prepared from commercially available 4-methoxybenzoic acid [55,56], furnished the sterically hindered biphenyls **7a**–**7d** in 48–50% yields.

*^a^* Reagents and conditions: (a) **8**, Ba(OH)_2_, DME/H_2_O, Pd(Ph_3_P)_4_, 80 °C, 6 h, 48% for **7a** from **9a**, 50% for **7b** from **9b**, 50% for **7c** from **9c**, and 50% for **7d** from **9d**; (b) BBr_3_, CH_2_Cl_2_, −78 °C, rt, 12 h, 92% for **23a** from **7a**, 90% for **23b** from **7b**, 91% for **23c** from **7c**, and 89% for **23d** from **7d**; (c) AcOH, reflux, 24 h, 89% for **3a** from **23a**, 90% for **3b** from **23b**, 91% for **3c** from **23c**, and 91% for **3d** from **23d**; (d) NaCN, DMSO, rt, 24 h, 72% for **3e** from **3a**, 69% for **3f** from **3b**, and 70% for **3g** from **3c**.

Exposure of **7a**–**7d** to boron tribromide led to trihydroxybiphenyl intermediates **23a**–**23d** in 89–92% yields, in which cleavage of all methyl ether groups and of the phenolic ether with introduction of the bromide in the case of **7a**–**7c** took place in a single step. Acetic acid mediated lactonization of polyphenols **23a**–**23d** afforded cannabilactones **3a**–**3d** in 89–91% yields. Subsequent exposure of **3a**–**3c** to sodium cyanide in dimethyl sulfoxide gave nitriles **3e**–**3g**.

### 2.2. Affinity for Cannabinoid Receptors

The abilities of the cannabilactone analogs **3a**–**3g** to displace the radiolabeled CB1/CB2 agonist CP-55,940 from membranes prepared from rat brain (a source of CB1) and HEK293 cells expressing mouse CB2 were determined as described earlier [38,57,58]. Inhibition constant values (*K*_i_) from the respective competition binding curves are listed in Table 1 in which our first generation cannabilactone analog AM1714 is included for comparison. The current data of AM1714 for mCB2 are slightly different when compared to those we published earlier [27]. This is because the compound was first assayed in a different mCB2 receptor preparation, e.g., mouse spleen membrane.

It should also be noted that the rat, mouse, and human CB1 receptors have 97–99% sequence identity across species and, as shown earlier (see for example [12,37,38,57]), are not expected to exhibit variations in their *K*_i_ values. However, the CB2 receptor shows less homology (~82%) between species than does CB1 (97–99%), and that variability could cause species-related differences in affinity. Indeed, in our original work on the cannabilactone class of compounds, we have identified species-specific variation in CB2 affinity [27]. For this reason, the key compounds were also assayed using membranes from HEK293 cells expressing human CB2 (hCB2). Data from the latter preparation are listed in Table 2.

As shown in Table 1, replacement of the C1′-*gem*-dimethyl group with the bulkier, more sterically confined cyclopentyl ring produces cannabilactone analogs with enhanced affinity and selectivity for the mCB2 relative to the rCB1 receptors. We also observe that this trend for mCB2 selectivity can be optimized by varying the length of the side chain and the substituent at the terminal carbon atom. Thus, analogs carrying five- to seven-atoms long side chains terminated with a bromine atom or a cyano group (**3c**, **3a**, **3b**, **3g**, and **3e**) exhibit 16- to 26-fold selectivity for mCB2 over rCB1. The more lipophilic C1′-*gem*-dimethyl-heptyl (**1b**) and C1′-cyclopentyl-heptyl (**3d**) analogs have comparable affinity (*K*_i_ = 4.7 ± 1.8 nM and 8.4 ± 2.5 nM) and selectivity (21- to 32-fold) for mCB2 over rCB1. Within these series, however, analog AM4346 with its longer chain (eight atoms) and C6′-cyano substituent has optimal properties: maximal binding affinity for mCB2 and minimal affinity for rCB1. In fact, AM4346 exhibits a remarkable 131-fold selectivity for mCB2 over rCB1.

The binding affinities for the human CB2 (hCB2) receptor of the three key analogs AM1714, AM4348, and AM4346 are listed in Table 2. We observe that although all analogs exhibit somewhat reduced affinity for hCB2 as compared to mCB2 (Table 1), the C1′-cyclopentyl-analogs show a pronounced reduction in the affinity differences between mCB2 and hCB2 as compared to C1′-*gem*-dimethyl analog. Thus, AM1714 has 18-fold greater affinity for mCB2 than for hCB2, but this preference is reduced to 3-fold for AM4348 and 7-fold for AM4346. With a roughly 19-fold preference, analog AM4346 has the highest selectivity for hCB2 over rCB1.

Synthesized as precursors to cannabilactones, binding affinities of the intermediate biphenyl compounds **23a**–**23d** for rCB1 and mCB2 were also determined (Table 3). To our surprise, these biphenyl analogs bind mCB2 with high affinity and substantial selectivity for that receptor over rCB1. In this series, the C1′-cyclopentyl-heptyl analog AM4347 has both the greatest binding affinity (*K*_i_ = 5.7 ± 1.5 nM) for mCB2 and the highest selectivity (60-fold) for that receptor over rCB1.

### 2.3. Functional Characterization

Functional characterization of three key cannabilactones (AM1710, AM1714, and AM4346) for the hCB2 receptor was carried out by measuring the decrease in forskolin stimulated cAMP, as detailed earlier [38,57]. Data are listed in Table 4 in which the standard cannabinoid agonist CP-55,940 is included for comparison. Our testing results show that all three compounds potently decreased the levels of cAMP, indicating that within this signaling mechanism these compounds behaved as potent agonists at the hCB2 receptor with the C1′-cyclopentyl-analog AM4346 being more potent (EC_50_ = 3.7 ± 1.5 nM, *E_(max)_* = 89%) than the C1′-*gem*-dimethyl-analogs AM1710 and AM1714 (EC_50_ = 10.5 ± 2.5 nM, *E_(max)_* = 73% and EC_50_ = 36.9 ± 6.8 nM, *E_(max)_* = 77% respectively).

In the same assay, one of the biphenyl analogs, compound **23d** (AM4347), failed to show any responses in concentrations up to 2 μM.

## 3. Experimental Section

Materials: all reagents and solvents were purchased from Aldrich Chemical Company, unless otherwise specified, and used without further purification. All anhydrous reactions were performed under a static argon atmosphere in flame-dried glassware using scrupulously dry solvents. Flash column chromatography employed silica gel 60 (230–400 mesh). All compounds were demonstrated to be homogeneous by analytical TLC on pre-coated silica gel TLC plates (Merck, 60 F_245_ on glass, layer thickness 250 μm), and chromatograms were visualized by phosphomolybdic acid staining. Melting points were determined on a micro-melting point apparatus and are uncorrected. IR spectra were recorded on a Perkin Elmer Spectrum One FT-IR spectrometer. NMR spectra were recorded in CDCl_3_, unless otherwise stated, on a Bruker Ultra Shield 400 WB plus (^1^H at 400 MHz, ^13^C at 100 MHz) or on a Varian INOVA-500 (^1^H at 500 MHz) spectrometers and chemical shifts are reported in units of δ relative to internal TMS. Multiplicities are indicated as br (broadened), s (singlet), d (doublet), t (triplet), q (quartet), m (multiplet) and coupling constants (*J*) are reported in hertz (Hz). Low and high-resolution mass spectra were performed in School of Chemical Sciences, University of Illinois at Urbana-Champaign. Mass spectral data are reported in the form of *m*/*z* (intensity relative to base = 100).

4-Bromo-3,5-dimethoxybenzyl chloride (13). To a stirred solution of 4-bromo-3,5-dimethoxyphenylmethanol (**12**) (4.74 g, 19.2 mmol) in anhydrous carbon tetrachloride (200 mL) at room temperature under an argon atmosphere was added triphenylphosphine (11.6 g, 42.2 mmol). The reaction mixture was refluxed for 45 min and then cooled to room temperature with spontaneous precipitation of triphenylphosphine oxide. To this suspension was added anhydrous hexane, the white precipitate was filtered off, washed with hexane, and the combined filtrate was evaporated under reduced pressure. The residue purified by flash column chromatography on silica gel (20% diethyl ether in petroleum ether) to give compound **13** (4.74 g, 93% yield) as a colorless oil which crystallized on standing. mp 65–67 °C (lit [44]. mp 69 °C); ^1^H NMR (500 MHz, CDCl_3_) δ 6.60 (s, 2H, 2-H, 6-H), 4.55 (s, 2H, -CH_2_Cl), 3.92 (s, 6H, OMe). The 4-bromo-3,5-dimethoxyphenylmethanol (**12**) was synthesized in two steps from commercially available 4-bromo-3,5-dihydroxybenzoic acid (**10**), following previously reported procedures [43].

4-Bromo-3,5-dimethoxyphenylacetonitrile (14). To a stirred suspension of sodium cyanide (2.9 g, 40.8 mmol) in DMSO (80 mL) at room temperature was added a solution of **13** (4.3 g, 16.2 mmol) in DMSO (80 mL) over a period of 10 min. The reaction mixture was stirred vigorously overnight and then diluted by adding ice, saturated aqueous NaCl solution and diethyl ether. The organic layer was separated, and the aqueous phase was extracted with diethyl ether. The combined organic layer was washed with brine, dried over MgSO_4_ and the solvent was evaporated under reduced pressure. The residue purified by flash column chromatography on silica gel (50 % diethyl ether in petroleum ether) to give **14** in 87% yield (3.6 g). mp 123–125°C (lit [44]. mp 125–126 °C); ^1^H NMR (500 MHz, CDCl_3_) δ 6.52 (s, 2H, 2-H, 6-H), 3.92 (s, 6H, OMe), 3.74 (s, 2H, -CH_2_CN).

1-(4-Bromo-3,5-dimethoxyphenyl)cyclopentanecarbonitrile (15). To a solution of **14** (3.42 g, 13.36 mmol) in anhydrous THF (120 mL), at 0 °C under an argon atmosphere, was added potassium bis(trimethylsilyl)amide (8 g, 40.2 mmol). The resulting slurry was stirred at the same temperature for 10 min, and then a solution of 1,4-dibromobutane (3.46 g, 16 mmol) in anhydrous THF (20 mL) was added dropwise. The reaction was stirred for an additional 10 min at 0 °C and then quenched by adding saturated aqueous NH_4_Cl solution (80 mL). The mixture was warmed to room temperature and diluted with diethyl ether (200 mL). The organic layer was separated and the aqueous phase extracted with diethyl ether. The combined organic layer was washed with brine and dried over MgSO_4_ and the solvent evaporated under reduced pressure. The residue was purified by flash column chromatography on silica gel (25% diethyl ether in hexane) to give **15** as a white solid in 70% yield (2.9 g). mp 135-138 °C; ^1^H NMR (500 MHz, CDCl_3_) δ 6.65 (s, 2H, 2-H, 6-H), 3.92 (s, 6H, OMe), 2.54–2.45 (m, 2H of the cyclopentane ring), 2.14–2.04 (m, 4H of the cyclopentane ring), 2.02–1.94 (m, 2H of the cyclopentane ring); mass spectrum *m*/*z* (relative intensity) 311 (M^+^+2, 99), 309 (M^+^, 100), 270 (80), 268 (81), 257 (6), 255 (6), 245 (7), 243, (7), 218 (10), 216 (10), exact mass calculated for C_14_H_16_BrNO_2_ 309.0364, found 309.0362.

1-(4-Bromo-3,5-dimethoxyphenyl)cyclopentanecarboxaldehyde (16). To a stirred solution of **15** (1.40 g, 4.52 mmol) in dry CH_2_Cl_2_ (50 mL), at −78 °C, under an argon atmosphere, was added diisobutylaluminum hydride (1 M solution in hexanes, 12 mL) dropwise. The reaction mixture was stirred at the same temperature for 1 h, and then quenched by the dropwise addition of potassium sodium tartrate (10% solution in water). The mixture was warmed to room temperature and stirred vigorously for 1 h. The organic layer was separated, and the aqueous phase extracted with diethyl ether. The combined organic layer was washed with brine, dried over MgSO_4_ and concentrated in vacuo. The crude product was purified by flash column chromatography on silica gel (25% diethyl ether in hexane) to give **16** as a white solid in 85% yield (1.2 g). mp 88–91 °C; ^1^H NMR (400 MHz, CDCl_3_) δ 9.39 (s, 1H, CHO), 6.44 (s, 2H, 2-H, 6-H), 3.90 (s, 6H, OMe), 2.56–2.47 (m, 2H of the cyclopentane ring), 1.95–1.86 (m, 2H of the cyclopentane ring), 1.84–1.73 (m, 2H of the cyclopentane ring), 1.72–1.62 (m, 2H of the cyclopentane ring); mass spectrum m/z (relative intensity), 314 (M++2, 20), 312 (M+, 21), 285 (100), 283 (100), 270 (6), 268 (6); exact mass calculated for C_14_H_17_BrO_3_ 312.0361, found 312.0361.

2-Bromo-1,3-dimethoxy-5-[1-(1,2-cis-4-phenoxy-buten-1-yl)cyclopentyl]benzene (17a). To a stirred suspension of 3-(phenoxypropyl)triphenylphosphonium bromide (2.30 g 4.82 mmol) in dry THF (50 mL) at 0 °C, under an argon atmosphere was added potassium bis(trimethylsilyl)amide (0.94 g, 4.72 mmol). To the resulting slurry was added a solution of **16** (0.30 g, 0.96 mmol) in dry THF (5 mL) dropwise. Stirring was continued for an additional 10 min at the same temperature and the reaction was quenched by adding saturated aqueous NH_4_Cl (20 mL). The mixture was warmed to room temperature and diluted with diethyl ether (40 mL). The organic layer was separated and the aqueous phase extracted with diethyl ether. The combined organic layer was washed with brine, dried over MgSO_4_ and the solvent evaporated under reduced pressure. The residue was chromatographed through a column of silica gel (10% diethyl ether in petroleum ether) to give **17a** as a colorless liquid in 94% yield (390 mg); ^1^H NMR (400 MHz, CDCl_3_) δ 7.23 (t, *J* = 8.1 Hz, 2H, 3-H, 5-H, OPh), 6.91 (t, *J* = 8.1 Hz, 1H, 4-H, OPh), 6.75 (d, *J* = 8.1 Hz, 2H, 2-H, 6-H, OPh), 6.61 (s, 2H, ArH), 5.90 (d, *J* = 11.3 Hz, 1H, 2′-H), 5.46 (dt, *J* = 11.3 Hz, *J* = 7.3 Hz, 1H, 3′-H), 3.86 (s, 6H, OMe), 3.74 (t, J = 6.6 Hz, 2H, 5′-H)), 2.20 (q, *J* = 6.8 Hz, 2H, 4′-H), 2.19-1.93 (m, 4H of the cyclopentane ring), 1.85–1.70 (m, 4H of the cyclopentane ring); mass spectrum *m*/*z* (relative intensity) 432 (M^+^+2, 68), 430 (M^+^, 67), 338 (62), 309 (35), 258 (65), 231 (90), 229 (90), 176 (52), 121 (100), 90 (78), 77 (72); exact mass calculated for C_23_H_27_BrO_3_ 430.1144, found 430.1146.

2-Bromo-1,3-dimethoxy-5-[1-(1,2-cis-5-phenoxy-penten-1-yl)cyclopentyl]benzene (17b). The synthesis was carried our as described for **17a** using (4-phenoxybutyl)triphenylphosphonium bromide (3.18 g, 6.47 mmol) in anhydrous THF (40 mL), potassium bis(trimethylsilyl)amide (1.72 g, 6.36 mmol), and a solution of **16** (0.42 g, 1.35 mmol) in anhydrous THF (5 mL). The crude product obtained after work up was chromatographed through a column of silica gel (10% diethyl ether in petroleum ether) to give **17b** as a colorless liquid in 93% yield (0.56 g). ^1^H NMR (400 MHz, CDCl_3_) δ 7.26 (t, *J* = 8.0 Hz, 2H, 3-H, 5-H, OPh), 6.91 (t, *J* = 8.0 Hz, 1H, 4-H, OPh), 6.79 (d, *J* = 8.0 Hz, 2H, 2-H, 6-H, OPh), 6.60 (s, 2H, ArH), 5.79 (d, *J* = 11.3 Hz, 1H, 2′-H), 5.37 (dt, *J* = 11.3 Hz, *J* = 7.3 Hz, 1H, 3′-H), 3.86 (s, 6H, OMe), 3.70 (t, J = 6.5 Hz, 2H, 6′-H), 2.05–1.87 (m, 6H, 4H of the cyclopentane ring and 4′-H, overlapping), 1.80-1.78 (m, 4H of the cyclopentane ring), 1.63 (qt, J = 6.9 Hz, 2H, 5′-H); mass spectrum *m*/*z* (relative intensity) 446 (M^+^+2, 9), 444 (M^+^, 9), 348 (12), 314 (21), 312 (21), 285 (99), 283 (100), 252 (12), 231 (25), 229 (25), 204 (44), 176 (27), 133 (8), 94 (48), 67 (49); exact mass calculated for C_24_H_29_BrO_3_ 444.1300, found 444.1301.

2-Bromo-1,3-dimethoxy-5-[1-(4-phenoxybutyl)cyclopentyl]benzene (9a). A mixture of **17a** (360 mg, 0.84 mmol) and 10% Pd/C (54 mg) in ethyl acetate (20 mL) was stirred vigorously under hydrogen atmosphere (room temperature overnight). The catalyst was removed by filtration through celite and the filtrate was evaporated under reduced pressure to give the product **9a** as a viscous liquid (346 mg, 95% yield) which was used in the next step without further purification. ^1^H NMR (400 MHz, CDCl_3_) δ 7.26 (t, *J* = 8.1 Hz, 2H, 3-H, 5-H, OPh), 6.91 (t, *J* = 8.1 Hz, 1H, 4-H, OPh), 6.83 (d, *J* = 8.1 Hz, 2H, 2-H, 6-H, OPh), 6.49 (s, 2H, ArH), 3.87 (s, 6H, OMe), 3.85 (t, *J* = 6.4 Hz, 2H, 5′-H), 1.95–1.88 (m, 2H of the cyclopentane ring), 1.87–1.79 (m, 2H of the cyclopentane ring), 1.77–1.59 (m, 8H, 4H of the cyclopentane ring and 4H of the 4-phenoxybutyl group), 1.22–1.14 (m, 2H of the 4-phenoxybutyl group); mass spectrum *m*/*z* (relative intensity) 434 (M^+^+2, 24), 432 (M^+^, 24), 342 (16), 340 (16), 285 (100), 283 (97), 231 (24), 229 (24), 204 (32), 176 (18), 149 (19), 91 (21), 67 (34); exact mass calculated for C_23_H_29_BrO_3_ 432.1300, found 432.1303.

2-Bromo-1,3-dimethoxy-5-[1-(5-phenoxypentlyl)cyclopentyl]benzene (9b). The title compound was synthesized as described for **9a**, using **17b** (550 mg, 1.26 mmol) and 10% Pd/C (80 mg) in EtOAc (20 mL) and gave **9b** as a white solid in 94% yield (529 mg). mp 65–67 °C; ^1^H NMR (500 MHz, CDCl_3_) δ 7.26 (t, *J* = 8.0 Hz, 2H, 3-H, 5-H, OPh), 6.92 (t, *J* = 8.0 Hz, 1H, 4-H, OPh), 6.84 (d, *J* = 8.0 Hz, 2H, 2-H, 6-H, OPh), 6.49 (s, 2H, ArH), 3.88 (s, 6H, OMe), 3.86 (t, *J* = 6.5 Hz, 2H, 6′-H), 1.94–1.86 (m, 2H of the cyclopentane ring), 1.85–1.77 (m, 2H of the cyclopentane ring), 1.75–1.63 (m, 6H, 4H of the cyclopentane ring and 2H of the 5-phenoxypentyl group, overlapping), 1.62–1.56 (m, 2H, 2′-H), 1.34 (qt, *J* = 7.6Hz, 2H of the 5-phenoxypentyl group), 1.10–1.00 (m, 2H of the 5-phenoxypentyl group); mass spectrum *m*/*z* (relative intensity) 448 (M++2, 7), 446 (M+, 8), 381 (5), 331 (6), 279 (12), 231 (14), 149 (55), 119 (34), 69 (100); exact mass calculated for C_24_H_31_BrO_3_ 446.1457, found 446.1457.

trans-3-[1-(4-Bromo-3,5-dimethoxyphenyl)cyclopentyl]acrylic acid ethyl ester (18). To a solution of triethyl phosphonoacetate (2.78 g, 12.42 mmol) in dry THF (50 mL), at 0 °C, under an argon atmosphere, was added sodium hydride (497 mg, 12.42 mmol, 60% dispersion in mineral oil). The mixture was stirred for 15 min at the same temperature and a solution of **16** (1.11 g, 3.55 mmol) in dry THF (10 mL) was added dropwise. Stirring was continued for an additional 10 min, and the reaction was quenched by adding saturated aqueous NH_4_Cl (20 mL). The mixture was warmed to room temperature and diluted with diethyl ether (100 mL). The organic phase was separated and the aqueous phase extracted with diethyl ether. The combined organic layer was washed with brine, dried over MgSO_4_ and evaporated under reduced pressure. The crude product was purified by flash column chromatography on silica gel (35% diethyl ether in hexane) to give **18** as a white solid in 92% yield (1.25 g). mp 68–72 °C; ^1^H NMR (400 MHz, CDCl_3_) δ 7.04 (d, *J* = 15.8 Hz, 1H, >CH=CH<), 6.48 (s, 2H, ArH), 5.62 (d, *J* = 15.8 Hz, 1H, >CH=CH<), 4.16 (q, *J* = 7.1 Hz, 2H, OC*H*_2_CH_3_), 3.88 (s, 6H, OMe), 2.18–2.08 (m, 2H of the cyclopentane ring), 2.07–1.96 (m, 2H of the cyclopentane ring), 1.84–1.72 (m, 4H of the cyclopentane ring), 1.27 (t, *J* = 7.1 Hz, 3H, OCH_2_C*H*_3_); mass spectrum m/z (relative intensity) 384 (M^+^+2, 100), 382 (M^+^, 100), 353 (16), 339 (22), 337 (22); exact mass calculated for C_18_H_23_BrO_4_ 382.0780, found 382.0778.

3-[1-(4-Bromo-3,5-dimethoxyphenyl)cyclopentyl]propionic acid ethyl ester (19a). A mixture of **18** (0.5 g, 1.3 mmol) and 10% Pd/C (100 mg) in ethyl acetate (20 mL) was placed in a Parr apparatus (Parr Instrument Co, Moline, IL) and treated with hydrogen at 30 psi for 6 h. The catalyst was removed by filtration through a pad of celite and the filtrate was evaporated under reduced pressure to give **19a** as a white solid (472 mg, 95% yield) which was used in the next step without further purification. mp 78–80 °C; ^1^H NMR (500 MHz, CDCl_3_) δ 6.48 (s, 2H, ArH), 4.03 (q, *J* = 7.1 Hz, 2H, OC*H*_2_CH_3_), 3.89 (s, 6H, OMe), 2.04–1.98 (m, 2H, 3′-H), 1.96–1.89 (m, 4H, 2H of the cyclopentane ring and 2′-H, overlapping), 1.86–1.65 (m, 6H of the cyclopentane ring), 1.20 (t, *J* = 7.1 Hz, 3H, OCH_2_C*H*_3_); mass spectrum *m*/*z* (relative intensity), 386 (M^+^+2, 16), 384 (M^+^, 15), 341 (8), 339 (8), 285 (37), 283 (36), 231 (8), 229 (8), 204 (15), 176 (9), 115 (7), 101 (9), 77 (20), 67 (100); exact mass calculated for C_18_H_25_BrO_4_ 384.0936, found 384.0934.

3-[1-(4-Bromo-3,5-dimethoxyphenyl)cyclopentyl]propionic acid methyl ester (19b). A mixture of **18** (0.7 g, 1.83 mmol) and magnesium turnings (132 mg, 5.5 mmol), in dry methanol (20 mL) was stirred at 0 °C for 2 h and at room temperature for an additional 12 h. The solvent was evaporated under reduced pressure and the residue diluted with water (20 mL) and diethyl ether (50 mL). To this mixture was added 5% aqueous HCl (10 mL), the organic layer was separated, and the aqueous layer extracted with diethyl ether. The combined ethereal layer was successively washed with NaHCO_3_ and brine, dried over MgSO_4_ and evaporated under reduced pressure to give the product **19b** as a white solid (543 mg, 80% yield) which was used in the next step without further purification. mp 71–74 °C; ^1^H NMR (500 MHz, CDCl_3_) δ 6.49 (s, 2H, ArH), 3.91 (s, 6H, OMe), 3.60 (s, 3H, COOCH_3_), 2.09–2.02 (m, 2H, 3′-H), 1.99–1.90 (m, 4H, 2H of the cyclopentane ring and 2′-H, overlapping), 1.88–1.65 (m, 6H, of the cyclopentane ring); mass spectrum *m*/*z* (relative intensity) 372 (M^+^+2, 98), 370 (M^+^, 100), 341 (19), 339 (20); exact mass calculated for C_17_H_23_BrO_4_ 370.0780, found 370.0783.

3-[1-(4-Bromo-3,5-dimethoxyphenyl)cyclopentyl]propan-1-ol (20). To a stirred solution of **19b** (520 mg, 1.4 mmol) in dry THF (20 mL), at room temperature, under an argon atmosphere, was added diisobutylaluminum hydride (3.7 mL, 1M solution in hexanes) over a period of 15 min. The mixture was stirred at the same temperature for 1 h, and then cooled to 0 °C, and the reaction was quenched by dropwise addition of aqueous potassium sodium tartrate (10% solution in water, 10 mL). The resulting mixture was warmed to room temperature, diluted with ethyl acetate (10 mL) and stirred vigorously for 1h. The organic layer was separated, and the aqueous phase extracted with ethyl acetate. The combined organic layer was washed with brine, dried over MgSO_4_ and concentrated in vacuo. The residue was purified by flash column chromatography on silica gel (40% ethyl acetate in hexane) to give **20** as a colorless liquid in 85% yield (408 mg). ^1^H NMR (500 MHz, CDCl_3_) δ 6.51 (s, 2H, ArH), 3.91 (s, 6H, OMe), 3.53 (t, *J* = 6.4 Hz, 2H, 4′-H), 1.98-1.91 (m, 2H of the cyclopentane ring), 1.89–1.83 (m, 2H of the cyclopentane ring), 1.81–1.63 (m, 6H, 4H of the cyclopentane ring and 2′-H, overlapping, especially 1.66, m, 2′-H), 1.34–1.26 (m, 2H, 3′-H); mass spectrum *m*/*z* (relative intensity) 342 (M^+^, 100), 297 (26), 285 (10), 283 (10); exact mass calculated for C_16_H_23_BrO_3_ 342.0831, found 342.0829. This compound was also synthesized by using **19a** (462 mg, 1.2 mmol) in anhydrous THF (10 mL) and diisobutylaluminum hydride (3 mL, 1M solution in hexanes) and gave 354 mg (86% yield) of product (**20**).

2-Bromo-1,3-dimethoxy-5-[1-(3-bromopropyl)cyclopentyl]benzene (21). To a stirred solution of **20** (400 mg, 1.17 mmol) and carbon tetrabromide (465 mg, 1.40 mmol) in anhydrous CH_2_Cl_2_ (10 mL) at 0 °C, under a nitrogen atmosphere, was added triphenylphosphine (368 mg, 1.40 mmol) portionwise. After the addition was completed, the reaction mixture was stirred for an additional 20 min, whereupon the solvent was removed in vacuo. The residue was purified by flash column chromatography on silica gel (10% diethyl ether in hexane) to give **21** as a colorless liquid in 85% yield (403 mg). ^1^H NMR (500 MHz, CDCl_3_) δ 6.48 (s, 2H, ArH), 3.89 (s, 6H, OMe), 3.27 (t, *J* = 6.4 Hz, 2H, 4′-H), 1.96–1.88 (m, 2H of the cyclopentane ring), 1.86–1.64 (m, 8H, 6H of the cyclopentane ring and 2′-H, overlapping), 1.60–1.52 (m, 2H, 3′-H); mass spectrum *m*/*z* (relative intensity) 408 (M^+^+4, 13), 406 (M^+^+2, 29), 404 (M^+^, 14), 327 (26), 325 (26), 285 (99), 283 (100), 233 (29), 231 (29), 204 (45), 289 (18), 176 (25), 109 (24), 67 (36); exact mass calculated for C_16_H_22_Br_2_O_2_ 403.9987, found 403.9987.

2-Bromo-1,3-dimethoxy-5-[1-(3-phenoxypropyl)cyclopentyl]benzene (9c). To a stirred solution of **21** (250 mg, 0.62 mmol) in DMSO (6 mL) at room temperature, under an argon atmosphere, was added sodium phenoxide trihydrate (530 mg, 3.10 mmol). The reaction mixture was stirred vigorously for 24 h and then diluted by adding ice water (5 mL) and diethyl ether. The organic layer was separated, and the aqueous phase was extracted with diethyl ether. The combined organic layer was washed with brine, dried over MgSO_4_ and evaporated under reduced pressure. Purification by flash column chromatography on silica gel (10% diethyl ether in hexane) gave **9c** as a colorless liquid in 50% yield (130 mg). ^1^H NMR (500 MHz, CDCl_3_) δ 7.24 (t, *J* = 7.5 Hz, 2H, 3-H, 5-H, OPh), 6.91 (t, *J* = 7.5 Hz, 1H, 4-H, OPh), 6.81 (d, *J* = 7.5 Hz, 2H, 2-H, 6-H, OPh), 6.51 (s, 2H, ArH), 3.89 (s, 6H, OMe), 3.80 (t, *J* = 6.5 Hz, 2H, 4′-H), 1.97-1.91 (m, 2H of the cyclopentane ring), 1.89–1.83 (m, 2H of the cyclopentane ring), 1.79–1.65 (m, 6H, 4H of the cyclopentane ring and 2′-H, overlapping), 1.53–1.46 (m, 2H, 3′-H); mass spectrum m/z (relative intensity) 420 (M^+^+2, 21), 418 (M^+^, 21), 327 (62), 325 (62), 285 (71), 283 (71), 231 (99), 229 (100), 204 (38), 151 (47), 109 (10), 67 (35); exact mass calculated for C_22_H_27_BrO_3_ 418.1144, found 418.1142.

2-Bromo-1,3-dimethoxy-5-(1-hexylcyclopentyl)benzene (9d). To a solution of **22** (1 g, 3.45 mmol) in anhydrous carbon tetrachloride (40 mL) at 0 °C, under an argon atmosphere was added bromine (7 mL, 1 M solution in CCl_4_). The reaction mixture was stirred at the same temperature for 20 min and the solvent was removed in vacuo. The residue was purified by flash column chromatography on silica gel (5% diethyl ether in hexane) to give **9d** as a white solid in 78% yield (0.99 g). mp 38–40 °C; ^1^H NMR (500 MHz, CDCl_3_) δ 6.49 (s, 2H, ArH), 3.89 (s, 6H, OMe), 1.91–1.85 (m, 2H of the cyclopentane ring), 1.84–1.76 (m, 2H of the cyclopentane ring), 1.75–1.60 (m, 4H of the cyclopentane ring), 1.58–1.50 (m, 2H, 2′-H), 1.25–1.09 (m, 6H, 4′-H, 5′-H, 6′-H), 1.01-0.93 (m, 2H, 3′-H), 0.83 (t, *J* = 7.2 Hz, 3H, 7′-H). mass spectrum *m*/*z* (relative intensity) 370 (M^+^+2, 30), 368 (M^+^, 30), 285 (100), 283 (98), 233 (20), 231 (20), 204 (36), 176 (21), 67 (25); exact mass calculated for C_19_H_29_BrO_2_ 368.1351, found 368.1351.

4′-[1-(4-Phenoxybutyl)cyclopentyl]-2′,5,6′-trimethoxy-N,N-diisopropyl-[1,1′-biphenyl]-2-carboxamide (7a). A degassed mixture of **9a** (135 mg, 0.31 mmol), boronic acid **8** (125 mg, 0.45 mmol), Ba(OH)_2_ (160 mg, 0.93 mmol), and Pd(PPh_3_)_4_ (70 mg, 0.06 mmol) in DME (5 mL)/water (1 mL) was flushed with argon, and heated in a sealed tube at 80 °C for 6 h with vigorous stirring. The reaction mixture was cooled to room temperature and diluted with EtOAc (30 mL) and the catalyst removed by filtration through celite. The filtrate was diluted with brine, and the organic layer was separated, dried (MgSO_4_), and concentrated in vacuo. Purification by flash column chromatography on silica gel (30% diethyl ether in hexane) gave **7a** as a viscous liquid in 48% yield (87 mg). IR (neat) 2927, 2857, 1625, 1607, 1576, 1337, 1241, 1128, 1032 cm^−1^; ^1^H NMR (400 MHz, CDCl_3_) δ 7.29 (t, *J* = 7.3 Hz, 2H, 3-H, 5-H, -OPh group), 7.27 (d, *J* = 7.2 Hz, 1H, 3-H, Ph-Ph group), 6.95 (t, *J* = 7.3 Hz, 1H, 4-H, -OPh group), 6.92–6.86 (d and dd overlapping, 3H, 2-H, 6-H of the -OPh group and 4-H of the Ph-Ph group), 6.82 (d, *J* = 2.5 Hz, 1H, 6-H, Ph-Ph group), 6.52 (s, 1H, 3′-H or 5′-H, Ph-Ph group), 6.51 (s, 1H, 5′-H or 3′-H, Ph-Ph group), 3.89 (t, *J* = 6.4 Hz, 2H, -C*H*_2_OPh), 3.84 (s, 3H, OMe), 3.74 (s, 3H, OMe), 3.73 (s, 3H, OMe), 3.71 (qt, *J* = 6.5 Hz, 1H, -C*H*(CH_3_)_2_), 3.16 (qt, *J* = 6.5 Hz, 1H, -C*H*(CH_3_)_2_), 2.08-1.97 (m, 2H of the cyclopentane ring), 1.91–1.60 (m, 10H, 6H of the cyclopentane ring and 4H of the 4-phenoxybutyl group, overlapping), 1.46 (d, *J* = 6.5 Hz, 3H, -CH(C*H*_3_)_2_), 1.38–1.25 (m, 2H of the 4-phenoxybutyl group), 1.10 (d, *J* = 6.5 Hz, 3H, -CH(C*H*_3_)_2_), 0.92 (d, *J* = 6.5 Hz, 3H, -CH(C*H*_3_)_2_), 0.58 (d, *J* = 6.5 Hz, 3H, -CH(C*H*_3_)_2_); mass spectrum m/z (relative intensity) 588 (M^+^+1, 22), 587 (M^+^, 54), 586 (29), 556 (10), 487 (84), 395 (100), 323 (12), 283 (15), 262 (44), 183 (20), 91 (11), 67 (9); exact mass calculated for C_37_H_49_NO_5_ 587.3611, found 587.3612.

4′-[1-(5-Phenoxypentyl)cyclopentyl]-2′,5,6′-trimethoxy-N,N-diisopropyl-[1,1′-biphenyl]-2-carboxamide (7b). The synthesis was carried out as described for **7a**, using **9b** (260 mg, 0.58 mmol), **8** (242 mg, 0.87 mol), Ba(OH)_2_ (300 mg, 1.74 mmol), and tetrakis(triphenylphosphine)palladium(0) (48 mg, 0.04 mmol) in DME (5 mL) and water (2 mL). The crude product obtained after workup was chromatographed through a column of silica gel (40% ethyl acetate in petroleum ether) to give **7b** as a viscous liquid in 50% yield (175 mg). IR (neat) 2924, 2855, 1624, 1602, 1572, 1335, 1241, 1127, 1030 cm^−1^; ^1^H NMR (400 MHz, CDCl_3_) δ 7.19 (t, *J* = 7.3 Hz, 2H, 3-H, 5-H, -OPh group), 7.18 (d, *J* = 7.0 Hz, 1H, 3-H, Ph-Ph group), 6.85 (t, *J* = 7.3 Hz, 1H, 4-H, -OPh group), 6.82–6.77 (d and dd overlapping, 3H, 2-H, 6-H of the -OPh group and 4-H of the Ph-Ph group), 6.72 (d, *J* = 2.6 Hz, 1H, 6-H, Ph-Ph group), 6.41 (s, 1H, 3′-H or 5′-H, Ph-Ph group), 6.40 (s, 1H, 5′-H or 3′-H, Ph-Ph group), 3.82 (t, *J* = 6.5 Hz, 2H, -C*H*_2_OPh), 3.73 (s, 3H, OMe), 3.64 (s, 3H, OMe), 3.63 (s, 3H, OMe), 3.61 (qt, *J* = 6.6 Hz, 1H, -C*H*(CH_3_)_2_), 3.06 (qt, *J* = 6.6 Hz, 1H, -C*H*(CH_3_)_2_), 1.95–1.83 (m, 2H of the cyclopentane ring), 1.81–1.51 (m, 10H, 6H of the cyclopentane ring and 4H of the 5-phenoxypentyl group, overlapping), 1.37 (d, *J* = 6.6 Hz, 3H, -CH(C*H*_3_)_2_), 1.25 (qt, *J* = 7.7 Hz, 2H of the 5-phenoxypentyl group), 1.10–0.95 (m and d overlapping, 5H, especially 1.03, d, *J* = 6.6 Hz, 3H, -CH(C*H*_3_)_2_), 0.82 (d, *J* = 6.6 Hz, 3H, -CH(C*H*_3_)_2_), 0.47 (d, *J* = 6.6 Hz, 3H, -CH(C*H*_3_)_2_); mass spectrum *m*/*z* (relative intensity) 602 (M^+^+1, 56), 601 (M^+^, 84), 600 (49), 570 (20), 530 (9), 501 (100), 407 (12), 323 (16), 297 (22), 269 (53), 257 (32), 95 (10), 67 (10); exact mass calculated for C_38_H_51_NO_5_ 601.3767, found 601.3765.

4′-[1-(3-Phenoxypropyl)cyclopentyl]-2′,5,6′-trimethoxy-N,N-diisopropyl-[1,1′-biphenyl]-2-carboxamide (7c). The synthesis was carried out as described for **7a**, using **9c** (90 mg, 0.21 mmol), **8** (108 mg, 0.39 mol), Ba(OH)_2_ (125 mg, 0.74 mmol) and tetrakis(triphenylphosphine)palladium(0) (48mg, 0.04mmol) in DME (5 mL) and water (1 mL). The crude product obtained after workup was chromatographed through a column of silica gel (40% ethyl acetate in petroleum ether) to give **7c** as a viscous liquid in 50% yield (60 mg). IR (neat) 2932, 2858, 1624, 1605, 1575, 1342, 1243, 1127, 1032 cm^−1^; ^1^H NMR (500 MHz, CDCl_3_) δ 7.26 (t, *J* = 7.7 Hz, 2H, 3-H, 5-H, -OPh group), 7.25 (d, *J* = 8.5 Hz, 1H, 3-H, Ph-Ph group), 6.92 (t, *J* = 7.7 Hz, 1H, 4-H, -OPh group), 6.87 (dd, *J* = 8.5 Hz, *J* = 3.0 Hz, 1H, 4-H of the Ph-Ph group), 6.83 (d, *J* = 7.7 Hz, 2H, 2-H, 6-H of the -OPh group), 6.79 (d, *J* = 3.0 Hz, 1H, 6-H, Ph-Ph group), 6.52 (s, 1H, 3′-H or 5′-H, Ph-Ph group), 6.50 (s, 1H, 5′-H or 3′-H, Ph-Ph group), 3.81 (t, *J* = 6.7 Hz, 2H, -C*H*_2_OPh), 3.80 (s, 3H, OMe), 3.72 (s, 3H, OMe), 3.71 (s, 3H, OMe), 3.69 (qt, *J* = 6.5 Hz, 1H, -C*H*(CH_3_)_2_), 3.14 (qt, *J* = 6.5 Hz, 1H, -C*H*(CH_3_)_2_), 2.04–1.94 (m, 2H of the cyclopentane ring), 1.88–1.81 (m, 2H of the cyclopentane ring), 1.80–1.65 (m, 6H, 4H of the cyclopentane ring and –C*H*_2_CH_2_CH_2_OPh, overlapping), 1.60–1.52 (m, 2H, –CH_2_C*H*_2_CH_2_OPh), 1.44 (d, *J* = 6.5 Hz, 3H, -CH(C*H*_3_)_2_), 1.07 (d, *J* = 6.5 Hz, 3H, -CH(C*H*_3_)_2_), 0.90 (d, *J* = 6.5 Hz, 3H, -CH(C*H*_3_)_2_), 0.57 (d, *J* = 6.5 Hz, 3H, -CH(C*H*_3_)_2_); mass spectrum *m*/*z* (relative intensity) 574 (M^+^+1, 23), 573 (M^+^, 65), 572 (36), 542 (13), 500 (11), 473 (82), 415 (19), 379 (22), 323 (13), 269 (100), 241 (23), 149 (32), 100 (27); exact mass calculated for C_36_H_47_NO_5_ 573.3454, found 573.3455.

4′-(1-Hexyl-cyclopentyl)-2′,5,6′-trimethoxy-*N*,*N*-diisopropyl-[1,1′-biphenyl]-2-carboxamide (7d). The synthesis was carried out as described for **7a**, using **9d** (90 mg, 0.21 mmol), **8** (108 mg, 0.39 mol), Ba(OH)_2_ (125 mg, 0.74 mmol), and tetrakis(triphenylphosphine)palladium(0) (48 mg, 0.04 mmol) in DME (5 mL) and water (1 mL). The crude product obtained after workup was chromatographed through a column of silica gel (40% ethyl acetate in petroleum ether) to give **7d** as a viscous liquid in 50% yield (60 mg). IR (neat) 2929, 2857, 1605, 1578, 1345 cm^−1^; ^1^H NMR (400 MHz, CDCl_3_) δ 7.26 (d, *J* = 8.4 Hz, 1H, 3-H, Ph-Ph group), 6.87 (dd, *J* = 8.4 Hz, *J* = 2.6 Hz, 1H, 4-H, Ph-Ph group), 6.79 (d, *J* = 2.6 Hz, 1H, 6-H, Ph-Ph group), 6.48 (s, 1H, 3′-H or 5′-H, Ph-Ph group), 6.47 (s, 1H, 5′-H or 3′-H, Ph-Ph group), 3.81 (s, 3H, OMe), 3.72 (s, 3H, OMe), 3.71 (s, 3H, OMe), 3.69 (qt, *J* = 6.5 Hz, 1H, -C*H*(CH_3_)_2_), 3.17 (qt, *J* = 6.5 Hz, 1H, -C*H*(CH_3_)_2_), 1.97–1.88 (m, 2H of the cyclopentane ring), 1.84–1.76 (m, 2H of the cyclopentane ring), 1.75-1.59 (m, 4H of the cyclopentane ring), 1.57–1.52 (m, 2H, 2′-H, hexyl group), 1.46 (d, *J* = 6.5 Hz, 3H, -CH(C*H*_3_)_2_), 1.30-1.13 (m, 6H, 4′-H, 5′-H, 6′-H, hexyl group), 1.09 (d, *J* = 6.5 Hz, 3H, -CH(C*H*_3_)_2_), 1.10-1.01 (m, 2H, 3′-H, hexyl group), 0.90 (d, *J* = 6.5 Hz, 3H, -CH(C*H*_3_)_2_), 0.87 (t, *J* = 7.1 Hz, 3H, 7′-H, hexyl group), 0.56 (d, *J* = 6.5 Hz, 3H, -CH(C*H*_3_)_2_); mass spectrum m/z (relative intensity) 524 (M^+^+1, 8), 523 (M^+^, 24) 522 (18), 492 (6), 450 (5), 423 (100), 337 (5), 269 (5), 235 (5), 192 (8), 135 (35), 94 (27), 57 (19); exact mass calculated for C_33_H_49_NO_4_ 523.3662, found 523.3664.

4′-[1-(4-Bromobutyl)cyclopentyl]-2′,5,6′-trihydroxy-N,N-diisopropyl-[1,1′-biphenyl]-2-carboxamide (23a). To a stirring solution of **7a** (80.0 mg, 0.14 mmol) in anhydrous CH_2_Cl_2_ (5 mL), at −78 °C, under an argon atmosphere, was added boron tribromide (0.7 mL, 1 M solution in CH_2_Cl_2_). The mixture was gradually warmed to room temperature and stirred for an additional 12 h. Unreacted boron tribromide was destroyed by the addition of methanol at 0 °C. The resulting mixture was warmed to room temperature and volatiles were removed in vacuo. The residue was diluted with ethyl acetate and washed with saturated NaHCO_3_ and brine. The organic layer was dried over MgSO_4_ and the solvent evaporated under reduced pressure. The crude product obtained after workup was purified by flash column chromatography on silica gel (50% diethyl ether in petroleum ether) to give **23a** as a white solid in 92% yield (68 mg). mp 183–185 °C; IR (neat) 3305 (br, OH), 2928, 2852, 1631, 1616, 1570, 1340 cm^−1^; ^1^H NMR (400 MHz, CDCl_3_) δ 7.92 (br s, 1H, OH), 7.12 (d, *J* = 8.1 Hz, 1H, 3-H, Ph-Ph group), 6.88 (dd, *J* = 8.1 Hz, *J* = 2.5 Hz, 1H, 4-H of the Ph-Ph group), 6.77 (d, *J* = 2.5 Hz, 1H, 6-H, Ph-Ph group), 6.54 (d, *J* = 1.2 Hz, 1H, 3′-H or 5′-H, Ph-Ph group), 6.46 (d, *J* = 1.2 Hz, 1H, 5′-H or 3′-H, Ph-Ph group), 6.19 (br s, 1H, OH), 4.54 (br s, 1H, OH), 3.79 (qt, *J* = 6.8 Hz, 1H, -C*H*(CH_3_)_2_), 3.30 (qt, *J* = 6.8 Hz, 1H, -C*H*(CH_3_)_2_), 3.26 (t, *J* = 6.5 Hz, 2H, -C*H*_2_Br), 1.93–1.85 (m, 2H of the cyclopentane ring), 1.76–1.53 (m, 10H, 6H of the cyclopentane ring and 4H of the 4-bromobutyl group, overlapping), 1.44 (d, *J* = 6.8 Hz, 3H, -CH(C*H*_3_)_2_), 1.30–1.21 (m, 2H of the 4-bromobutyl group), 1.07 (d, *J* = 6.8 Hz, 3H, -CH(C*H*_3_)_2_), 1.06 (d, *J* = 6.8 Hz, 3H, -CH(C*H*_3_)_2_), 0.86 (d, *J* = 6.8 Hz, 3H, -CH(C*H*_3_)_2_); mass spectrum *m*/*z* (relative intensity) 533 (M^+^+2, 7), 531 (M^+^, 7), 451 (16), 433 (10), 431 (10), 351 (18), 295 (21), 268 (8), 241 (12), 102 (32), 86 (100); exact mass calculated for C_28_H_38_BrNO_4_ 531.1984, found 531.1980.

4′-[1-(5-Bromopentyl)cyclopentyl]-2′,5,6′-trihydroxy-N,N-diisopropyl-[1,1′-biphenyl]-2-carboxamide (23b). The synthesis was carried out as described for **23a**, using **7b** (165 mg, 0.27 mmol) and BBr_3_ (1.7 mL, 1M solution in CH_2_Cl_2_) in anhydrous CH_2_Cl_2_ (5 mL). The crude product obtained after workup was purified by flash column chromatography on silica gel (50% diethyl ether in petroleum ether) to give **23b** as a white solid in 90% yield (130 mg). mp 190–192 °C; IR (neat) 3312 (br, OH), 2925, 2857, 1632, 1615, 1567, 1343 cm^−1^; ^1^H NMR (400 MHz, CDCl_3_) δ 8.16 (br s, 1H, OH), 7.11 (d, *J* = 8.4 Hz, 1H, 3-H, Ph-Ph group), 6.84 (dd, *J* = 8.4 Hz, *J* = 2.7 Hz, 1H, 4-H of the Ph-Ph group), 6.68 (d, *J* = 2.7 Hz, 1H, 6-H, Ph-Ph group), 6.59 (d, *J* = 1.3 Hz, 1H, 3′-H or 5′-H, Ph-Ph group), 6.47 (d, *J* = 1.3 Hz, 1H, 5′-H or 3′-H, Ph-Ph group), 6.37 (br s, 1H, OH), 4.74 (br s, 1H, OH), 3.70 (qt, *J* = 7.0 Hz, 1H, -C*H*(CH_3_)_2_), 3.34 (qt, *J* = 7.0 Hz and t *J* = 6.8 Hz overlapping, 3H, -C*H*(CH_3_)_2_, -C*H*_2_Br), 1.94–1.85 (m, 2H of the cyclopentane ring), 1.79–1.52 (m, 10H, 6H of the cyclopentane ring and 4H of the 5-bromopentyl group, overlapping), 1.46 (d, *J* = 7.0 Hz, 3H, -CH(C*H*_3_)_2_), 1.28 (qt, *J* = 6.9 Hz, 2H of the 5-bromopentyl group), 1.07 (d, *J* = 7.0 Hz, 3H, -CH(C*H*_3_)_2_), 1.06 (d, *J* = 7.0 Hz, 3H, -CH(C*H*_3_)_2_), 1.05–0.97 (m, 2H of the 5-bromopentyl group), 0.87 (d, *J* = 7.0 Hz, 3H, -CH(C*H*_3_)_2_); mass spectrum m/z (relative intensity) 547 (M^+^+2, 8), 545 (M^+^, 8), 465 (8), 447 (17), 445 (16), 367 (8), 335 (16), 295 (23), 267 (8), 241 (15), 101 (24), 86 (100); exact mass calculated for C_29_H_40_BrNO_4_ 545.2141, found 545.2145.

4′-[1-(3-Bromopropyl)cyclopentyl]-2′,5,6′-trihydroxy-N,N-diisopropyl-[1,1′-biphenyl]-2-carboxamide (23c). The synthesis was carried out as described for **23a**, using **7c** (52 mg, 0.09 mmol) and BBr_3_ (0.6 mL, 1M solution in CH_2_Cl_2_) in anhydrous CH_2_Cl_2_ (5 mL). The crude product obtained after workup was purified by flash column chromatography on silica gel (50% diethyl ether in petroleum ether) to give **23c** as a white solid in 91% yield (42 mg). mp 178–180 °C; IR (neat) 3312 (br, OH), 2924, 2855, 1630, 1617, 1572, 1335 cm^−1^; ^1^H NMR (500 MHz, CDCl_3_) δ 8.14 (br s, 1H, OH), 7.13 (d, *J* = 8.5 Hz, 1H, 3-H, Ph-Ph group), 6.86 (dd, *J* = 8.5 Hz, *J* = 2.7 Hz, 1H, 4-H of the Ph-Ph group), 6.65 (d, *J* = 2.7 Hz, 1H, 6-H, Ph-Ph group), 6.60 (d, *J* = 1.5 Hz, 1H, 3′-H or 5′-H, Ph-Ph group), 6.48 (d, *J* = 1.5 Hz, 1H, 5′-H or 3′-H, Ph-Ph group), 6.30 (br s, 1H, OH), 4.72 (br s, 1H, OH), 3.71 (qt, *J* = 7.0 Hz, 1H, -C*H*(CH_3_)_2_), 3.33 (qt, *J* = 7.0 Hz, 1H, -C*H*(CH_3_)_2_), 3.23 (t, *J* = 6.5 Hz, 2H, -C*H*_2_Br), 1.98–1.88 (m, 2H of the cyclopentane ring), 1.78–1.62 (m, 8H, 6H of the cyclopentane ring and -C*H*_2_CH_2_CH_2_Br, overlapping), 1.59–1.53 (m, 2H, -CH_2_C*H*_2_CH_2_Br), 1.45 (d, *J* = 7.0 Hz, 3H, -CH(C*H*_3_)_2_), 1.07 (d, *J* = 7.0 Hz, 6H, -CH(C*H*_3_)_2_), 0.87 (d, *J* = 7.0 Hz, 3H, -CH(C*H*_3_)_2_); mass spectrum m/z (relative intensity) 519 (M^+^+2, 4), 517 (M^+^, 4), 437 (28), 336 (56), 296 (22), 294 (24), 241 (9), 102 (43), 86 (100); exact mass calculated for C_27_H_36_BrNO_4_ 517.1828, found 517.1830.

4′-(1-Hexyl-cyclopentyl)-2′,5,6′-trihydroxy-N,N-diisopropyl-[1,1′-biphenyl]-2-carboxamide (23d). The synthesis was carried out as described for **23a**, using **7d** (57 mg, 0.11 mmol) and BBr_3_ (0.7 mL, 1M solution in CH_2_Cl_2_) in anhydrous CH_2_Cl_2_ (5 mL). The crude product obtained after workup was purified by flash column chromatography on silica gel (50% diethyl ether in petroleum ether) to give **23d** as a white solid in 89% yield (47 mg). mp 185–187 °C; IR (neat) 3295 (br, OH), 2925, 2858, 1632, 1617, 1565, 1340 cm^−1^; ^1^H NMR (400 MHz, CDCl_3_) δ 8.00 (br s, 1H, OH), 7.15 (d, *J* = 8.5 Hz, 1H, 3-H, Ph-Ph group), 6.89 (dd, *J* = 8.5 Hz, *J* = 2.7 Hz, 1H, 4-H of the Ph-Ph group), 6.75 (d, *J* = 2.7 Hz, 1H, 6-H, Ph-Ph group), 6.60 (d, *J* = 1.5 Hz, 1H, 3′-H or 5′-H, Ph-Ph group), 6.47 (d, *J* = 1.5 Hz, 1H, 5′-H or 3′-H, Ph-Ph group), 5.63 (br s, 1H, OH), 4.64 (br s, 1H, OH), 3.71 (qt, *J* = 7.0 Hz, 1H, -C*H*(CH_3_)_2_), 3.31 (qt, *J* = 7.0 Hz, 1H, -C*H*(CH_3_)_2_), 1.95-1.85 (m, 2H of the cyclopentane ring), 1.78–1.59 (m, 6H of the cyclopentane ring), 1.57–1.49 (m, 2H, 2′-H), 1.47 (d, *J* = 7.0 Hz, 3H, -CH(C*H*_3_)_2_), 1.25–1.10 (m, 6H, 4′-H, 5′-H, 6′-H), 1.08 (d, *J* = 7.0 Hz, 3H, -CH(C*H*_3_)_2_), 1.07 (d, *J* = 7.0 Hz, 3H, -CH(C*H*_3_)_2_), 1.04-0.97 (m, 2H, 3′-H), 0.87 (d, *J* = 7.0 Hz, 3H, -CH(C*H*_3_)_2_), 0.82 (t, *J* = 7.2 Hz, 3H, 7′-H). mass spectrum *m*/*z* (relative intensity) 482 (M^+^+1, 15), 481 (M^+^, 37), 86 (100); exact mass calculated for C_30_H_43_NO_4_ 481.3192, found 481.3191.

3-[1-(4-Bromobutyl)cyclopentyl]-1,9-dihydroxy-6H-dibenzo[b,d]pyran-6-one (3a). A stirred solution of **23a** (50 mg, 0.094 mmol) in glacial acetic acid (4 mL) and water (1 mL) was refluxed for 24 h. The reaction mixture was cooled to room temperature and diluted with aqueous NaHCO_3_ solution (5 mL) and ethyl acetate (20 mL). The organic phase was separated, and the aqueous phase extracted with ethyl acetate. The combined organic layer was washed with brine, dried over MgSO_4_ and concentrated in vacuo. The residue was purified by flash column chromatography on silica gel (60% diethyl ether in hexanes) to give **3a** as a white solid in 89%yield (36 mg). mp 96–98 °C; IR (neat) 3272 (br, OH), 2941, 1670 (>C=O), 1611, 1272, 1110 cm^−1^; ^1^H NMR (400 MHz, CDCl_3_) δ 8.45 (d, *J* = 2.4 Hz, 1H, 10-H), 8.33 (d, *J* = 8.7 Hz, 1H, 7-H), 7.01 (dd, *J* = 8.7 Hz, *J* = 2.4 Hz, 1H, 8-H), 6.88 (d, *J* = 1.6 Hz, 1H, 4-H or 2-H), 6.64 (d, *J* = 1.6 Hz, 1H, 2-H or 4-H), 6.32–5.50 (br s, 2H, OH), 3.29 (t, *J* = 6.8 Hz, 2H, -C*H*_2_Br), 1.97–1.87 (m, 2H of the cyclopentane ring), 1.86–1.51 (m, 10H, 6H of the cyclopentane ring and 4H of the 4-bromobutyl group, overlapping), 1.21–1.10 (m, 2H of the 4-bromobutyl group); ^13^C NMR (100 MHz, CD_3_OD) δ 165.2, 163.9, 157.7, 154.0, 153.0, 138.9, 133.3, 117.0, 113.9, 113.1, 111.6, 107.9, 105.8, 52.4, 42.0, 38.7, 34.5, 34.2, 25.2, 24.2; mass spectrum *m*/*z* (relative intensity) 432 (M^+^+2, 8), 430 (M^+^, 8), 350 (11), 321 (33), 295 (100), 267 (15), 91(3). exact mass calculated for C_22_H_23_BrO_4_ 430.0780, found 430.0778.

3-[1-(5-Bromopentyl)cyclopentyl]-1,9-dihydroxy-6H-dibenzo[b,d]pyran-6-one (3b). The synthesis was carried out as described for **3a** using **23b** (55 mg, 0.10 mmol) in glacial acetic acid (4 mL) and water (1 mL) and gave **3b** as a white solid in 90% yield (40 mg). mp 101-103 °C; IR (neat) 3275 (br, OH), 2923, 1668 (>C=O), 1607, 1272, 1105 cm^−1^; ^1^H NMR (500 MHz, CDCl_3_) δ 8.37 (d, *J* = 2.1 Hz, 1H, 10-H), 8.27 (d, *J* = 8.3 Hz, 1H, 7-H), 6.94 (dd, *J* = 8.3 Hz, *J* = 2.1 Hz, 1H, 8-H), 6.81 (d, *J* = 1.1 Hz, 1H, 4-H or 2-H), 6.56 (d, *J* = 1.1 Hz, 1H, 2-H or 4-H), 6.02 (br s, 1H, OH), 5.87 (br s, 1H, OH), 3.24 (t, *J* = 6.7 Hz, 2H, -C*H*_2_Br), 1.89–1.80 (m, 2H of the cyclopentane ring), 1.76–1.48 (m, 10H, 6H of the cyclopentane ring and 4H of the 5-bromopentyl group, overlapping), 1.22 (qt, *J* = 6.5 Hz, 2H of the 5-bromopentyl group), 1.01–0.89 (m, 2H of the 5-bromopentyl group); ^13^C NMR (100 MHz, CD_3_OD) δ 165.2, 163.9, 157.7, 154.0, 153.2, 138.9, 133.3, 116.9, 113.9, 113.1, 111.6, 107.9, 105.8, 52.4, 42.8, 38.7, 34.3, 33.9, 29.8, 25.8, 24.2; mass spectrum *m*/*z* (relative intensity) 446 (M^+^+2, 10), 444 (M^+^, 10), 364 (11), 335 (28), 295 (100), 267 (16), 241 (42), 91(5), 67 (14); exact mass calculated for C_23_H_25_BrO_4_ 444.0936, found 444.0930.

3-[1-(3-Bromopropyl)cyclopentyl]-1,9-dihydroxy-6H-dibenzo[b,d]pyran-6-one (3c). The synthesis was carried out as described for **3a** using **23c** (30 mg, 0.058 mmol) in glacial acetic acid (4 mL) and water (1 mL) and gave **3c** as a white solid in 91% yield (22 mg). mp 105–106 °C; IR (neat) 3285 (br, OH), 2928, 1668 (>C=O), 1610, 1272, 1110 cm^−1^; ^1^H NMR (500 MHz, CDCl_3_) δ 8.49 (d, *J* = 2.0 Hz, 1H, 10-H), 8.32 (d, *J* = 8.3 Hz, 1H, 7-H), 7.00 (dd, *J* = 8.3 Hz, *J* = 2.0 Hz, 1H, 8-H), 6.67 (d, *J* = 1.4 Hz, 1H, 4-H or 2-H), 6.52 (d, *J* = 1.4 Hz, 1H, 2-H or 4-H), 6.35–5.89 (br s, 2H, OH), 3.25 (t, *J* = 6.5 Hz, 2H, -C*H*_2_Br), 1.96–1.86 (m, 2H of the cyclopentane ring), 1.77–1.60 (m, 8H, 6H of the cyclopentane ring and -C*H*_2_CH_2_CH_2_Br, overlapping), 1.57–1.52 (m, 2H, -CH_2_C*H*_2_CH_2_Br), mass spectrum m/z (relative intensity) 418 (M^+^+2, 6), 416 (M^+^, 6), 336 (9), 307 (25), 295 (100), 267 (18), 91(3); exact mass calculated for C_21_H_21_BrO_4_ 416.0623, found 416.0627.

3-(1-Hexyl-cyclopentyl]-1,9-dihydroxy-6H-dibenzo[b,d]pyran-6-one (3d). The synthesis was carried out as described for **3a**, using **23d** (40 mg, 0.083 mmol) in glacial acetic acid (4 mL) and water (1 mL), and gave **3d** as a white solid in 91% yield (28 mg). mp 153–155 °C; IR (neat) 3278 (br, OH), 2925, 1667 (>C=O), 1614, 1384, 1275, 1108 cm^−1^; ^1^H NMR (500 MHz, CDCl_3_) δ 8.41 (d, *J* = 2.4 Hz, 1H, 10-H), 8.33 (d, *J* = 8.7 Hz, 1H, 7-H), 7.00 (dd, *J* = 8.7 Hz, *J* = 2.4 Hz, 1H, 8-H), 6.90 (d, *J* = 1.6 Hz, 1H, 4-H or 2-H), 6.61 (d, *J* = 1.6 Hz, 1H, 2-H or 4-H), 5.65 (br s, 1H, OH), 5.50 (br s, 1H, OH), 1.94–1.87 (m, 2H of the cyclopentane ring), 1.82–1.62 (m, 6H of the cyclopentane ring), 1.60–1.52 (m, 2H, 2′-H), 1.24–1.12 (m, 6H, 4′-H, 5′-H, 6′-H), 1.03–0.94 (m, 2H, 3′-H), 0.82 (t, *J* = 6.9 Hz, 3H, 7′-H); ^13^C NMR (100 MHz, CD_3_OD) δ 165.2, 164.0, 157.7, 153.9, 153.3, 139.0, 133.3, 116.9, 113.9, 113.0, 111.7, 107.9, 105.7, 52.5, 43.0, 38.8, 32.9, 31.0, 26.5, 24.3, 23.7, 14.4; mass spectrum *m*/*z* (relative intensity) 381 (M^+^+1, 6), 380 (M^+^, 18), 295 (4), 270 (23), 241 (11), 205 (71), 149 (62), 135 (80), 91 (63), 83 (54), 69 (37), 59 (100); exact mass calculated for C_24_H_28_O_4_ 380.1988, found 380.1986.

3-[1-(4-Cyanobutyl)cyclopentyl]-1,9-dihydroxy-6H-dibenzo[b,d]pyran-6-one (3e). To a stirring solution of **3a** (30 mg, 0.07 mmol) in anhydrous DMSO (5 mL), at room temperature, under an argon atmosphere, was added and NaCN (17 mg, 0.35 mmol). The reaction mixture was stirred vigorously for 24 h and then diluted by adding ice water (2 mL) and diethyl ether (10 mL). The organic layer was separated, and the aqueous phase was extracted with diethyl ether (2 × 5 mL). The combined organic layer was washed with brine, dried over MgSO_4_ and concentrated under reduced pressure. The crude material was purified by flash column chromatography on silica gel (40% ethyl acetate in petroleum ether) gave **3e** as a white solid in 72% yield (19 mg). mp 138–140 °C; IR (neat) 3275 (br, OH), 2925, 2853, 2259 (w, C≡N), 1684 (>C=O), 1602, 1270, 1107 cm^−1^; ^1^H NMR (400 MHz, CDCl_3_) δ 9.82 (br s, 1H, OH), 9.78 (br s, 1H, OH), 8.60 (d, *J* = 2.3 Hz, 1H, 10-H), 8.22 (d, *J* = 8.7 Hz, 1H, 7-H), 6.98 (dd, *J* = 8.7 Hz, *J* = 2.3 Hz, 1H, 8-H), 6.78 (d, *J* = 1.7 Hz, 1H, 4-H or 2-H), 6.77 (d, *J* = 1.7 Hz, 1H, 2-H or 4-H), 2.24 (t, *J* = 7.1 Hz, 2H, -C*H*_2_CN), 1.99–1.89 (m, 2H of the cyclopentane ring), 1.80–1.57 (m, 8H, 6H of the cyclopentane ring and 2H of the 4-cyanobutyl group, overlapping), 1.53 (qt, *J* = 7.6 Hz, 2H of the 4-cyanobutyl group), 1.23–1.11 (m, 2H of the 4-cyanobutyl group); mass spectrum *m*/*z* (relative intensity) 378 (M^+^+1, 8), 377 (M^+^, 27), 368 (5), 309 (5), 295 (100), 279 (98), 241 (27), 224 (31), 205 (20), 91 (13), 67 (14); exact mass calculated for C_23_H_23_NO_4_ 377.1627, found 377.1625.

3-[1-(5-Cyanopentyl)cyclopentyl]-1,9-dihydroxy-6H-dibenzo[b,d]pyran-6-one (3f). The synthesis was carried out as described for **3e**, using **3b** (31 mg, 0.07 mmol) and NaCN (17 mg, 0.35 mmol) in DMSO (5 mL), and gave **3f** as a white solid in 69% yield (19 mg). mp 176–178 °C; IR (neat) 3281 (br, OH), 2923, 2854, 2259 (w, C≡N), 1683 (>C=O), 1618, 1598, 1271, 1216, 1103 cm^−1^; ^1^H NMR (500 MHz, CDCl_3_) δ 8.45 (d, *J* = 2.0 Hz, 1H, 10-H), 8.33 (d, *J* = 8.2 Hz, 1H, 7-H), 7.02 (dd, *J* = 8.2 Hz, *J* = 2.0 Hz, 1H, 8-H), 6.88 (d, *J* = 1.2 Hz, 1H, 4-H or 2-H), 6.65 (d, *J* = 1.2 Hz, 1H, 2-H or 4-H), 6.20 (br s, 1H, OH), 5.87 (br s, 1H, OH), 2.26 (t, *J* = 6.8 Hz, 2H, -C*H*_2_CN), 1.97-1.90 (m, 2H of the cyclopentane ring), 1.81–1.52 (m, 10H, 6H of the cyclopentane ring and 4H of the 5-cyanopentyl group, overlapping), 1.35 (qt, *J* = 7.3 Hz, 2H of the 5-cyanopentyl group), 1.10–1.03 (m, 2H of the 5-cyanopentyl group); ^13^C NMR (100 MHz, CD_3_OD) δ 165.2, 163.9, 157.7, 154.0, 153.1, 138.9, 133.3, 121.2, 116.9, 113.9, 113.1, 111.6, 107.9, 105.8, 52.4, 42.7, 38.7, 30.4, 26.4, 25.9, 24.2, 17.3; mass spectrum *m*/*z* (relative intensity) 392 (M^+^+1, 15), 391 (M^+^, 44), 349 (4), 306 (100), 295 (15), 266 (11), 252 (28), 240 (12), 190 (4), 149 (6), 67 (10); exact mass calculated for C_24_H_25_NO_4_ 391.1784, found 391.1781.

3-[1-(3-Cyanopropyl)cyclopentyl]-1,9-dihydroxy-6H-dibenzo[b,d]pyran-6-one (3g). The synthesis was carried out as described for **3e**, using **3c** (18 mg, 0.043 mmol) and NaCN (11 mg, 0.22 mmol) in DMSO (5 mL) and gave **3g** as a white solid in 70% yield (13 mg). mp 118–120 °C; IR (neat) 3285 (br, OH), 2928, 2853, 2260 (w, C≡N), 1683 (>C=O), 1599, 1272, 1105 cm^−1^; ^1^H NMR (500 MHz, CDCl_3_) δ 8.47 (d, *J* = 2.1 Hz, 1H, 10-H), 8.31 (d, *J* = 8.3 Hz, 1H, 7-H), 6.98 (dd, *J* = 8.3 Hz, *J* = 2.1 Hz, 1H, 8-H), 6.64 (d, *J* = 1.3 Hz, 1H, 4-H or 2-H), 6.49 (d, *J* = 1.3 Hz, 1H, 2-H or 4-H), 6.22 (br s, 1H, OH), 5.78 (br s, 1H, OH), 2.23 (t, *J* = 6.7 Hz, 2H, -C*H*_2_CN), 1.94-1.83 (m, 2H of the cyclopentane ring), 1.74–1.56 (m, 8H, 6H of the cyclopentane ring and -C*H*_2_CH_2_CH_2_CN, overlapping), 1.55–1.49 (m, 2H, -CH_2_C*H*_2_CH_2_CN); mass spectrum m/z (relative intensity) 364 (M^+^+1, 7), 363 (M^+^, 22), 336 (67), 309 (24), 295 (100), 279 (48), 241 (47), 165 (10), 129 (24); exact mass calculated for C_22_H_21_NO_4_ 363.1471, found 363.1470.

Radioligand binding assays: the affinities (*K*_i_) of the new compounds for rat CB1 receptor as well as for mouse and human CB2 receptors were obtained by using membrane preparations from rat brain or HEK293 cells expressing either mCB2 or hCB2 receptors, respectively, and [^3^H]CP-55,940 as the radioligand, as previously described [38,57]. Results from the competition assays were analyzed using nonlinear regression to determine the IC_50_ values for the ligand [59]; *K*_i_ values were calculated from the IC_50_ (Prism by GraphPad Software, Inc.). Each experiment was performed in triplicate and *K*_i_ values determined from three independent experiments and are expressed as the mean of the three values.

cAMP assay: [57,60] HEK293 cells stably expressing hCB2 receptors were used for the studies. The cAMP assay was carried out using PerkinElmer’s Lance ultra cAMP kit following the protocol of the manufacturer. Briefly, the assays were carried out in 384-well plates using 1000–1500 cells/well. The cells were harvested with non-enzymatic cell dissociation reagent Versene, washed once with HBSS and resuspended in the stimulation buffer. The various concentrations of the test compound (5 μL) in forskolin (2 μM final concentration) containing stimulation buffer were added to the plate followed by the cell suspension (5 μL). Cells were stimulated for 30 min at room temperature. Eu-cAMP tracer working solution (5 μL) and Ulight-anti-cAMP working solution (5 μL) were then added to the plate and incubated at room temperature for 60 min. The data were collected on a Perkin-Elmer Envision instrument. The EC_50_ values were determined by non-linear regression analysis using GraphPad Prism software (GraphPad Software, Inc., San Diego, CA, USA).

## 4. Conclusions

This study expands on earlier work on the CB2-selective cannabilactone prototype. We explored the effects of modifications to the pharmacophoric side chain. In particular, we replaced the C1′-*gem*-dimethyl group in AM1714 with the bulkier cyclopentyl ring, varied the chain’s length, and substituted a bromo or cyano group for the terminal carbon. We identified the analog 6-[1-(1,9-dihydroxy-6-oxo-6*H*-benzo[*c*]chromen-3-yl)cyclopentyl]hexanenitrile (AM4346) as a high-affinity ligand to mCB2 (*K*_i_ = 4.9 nM) with 131-fold selectivity for mCB2 over rCB1. Moreover, the species difference in the affinities of AM4346 between the mouse and the human CB2 receptors is reduced when compared to our first-generation compound AM1714. Importantly, in the cyclase assay AM4346 was found to be a highly potent and efficacious hCB2 receptor agonist (EC_50_ = 3.7 ± 1.5 nM, *E_(max)_* = 89%). Our lead cannabilactone analog, AM4346, is also endowed with enhanced polarity due to the incorporation of the cyano group. Extension of our SAR to include the biphenyl synthetic intermediates has revealed new compounds that bind mCB2 with high affinity and substantial selectivity for that receptor over rCB1.

## Figures and Tables

**Table 1:**
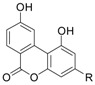
Affinities of side chain-modified cannabilactone analogs for rCB1 and mCB2 cannabinoid receptors (± 95% confidence limits).

Compound	R	*K*_i_ (nM) ^1^		rCB1/mCB2
		rCB1	mCB2	
**1b** **AM1714**	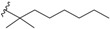	100 ± 24	4.7 ± 1.8	21.3
**3c** **AM4354**	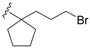	405 ± 122	24.3 ± 3.9	16.7
**3a** **AM4350**	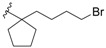	390 ± 132	20.8 ± 5.9	18.8
**3b** **AM4345**	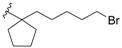	370 ± 130	16.3 ± 5.2	22.7
**3g** **AM4355**	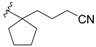	290 ± 64	17.9 ± 5.8	16.2
**3e** **AM4351**	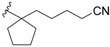	304 ± 73	11.8 ± 4.1	25.7
**3f** **AM4346**	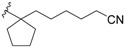	640 ± 152	4.9 ± 1.2	130.6
**3d** **AM4348**	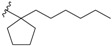	266 ± 58	8.4 ± 2.5	31.7

^1^ Affinities were determined using rat brain (CB1) or membranes from HEK293 cells expressing mouse CB2 and [^3^H]CP-55,940 as the radioligand following previously described procedures [38,57,58]. Data were analyzed using nonlinear regression analysis. *K*_i_ values were obtained from three independent experiments performed in triplicate and are expressed as the mean of the three values.

**Table 2:**
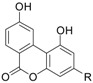
Affinities of key cannabilactone analogs for hCB2 cannabinoid receptors (± 95% confidence limits).

Compound	R	*K*_i_ (nM) ^1^		rCB1/hCB2	hCB2/mCB2
		rCB1	hCB2		
**1b** **AM1714**	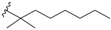	100 ± 24	83.6 ± 23.2	1.2	17.7
**3f** **AM4346**	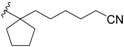	640 ± 152	33.8 ± 10.4	18.9	6.9
**3d** **AM4348**	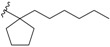	266 ± 58	28.0 ± 9.7	9.5	3.3

^1^ Cannabinoid receptors were prepared as described for Table 1, except that hCB2 was from HEK293 expressing the *human* and not the mouse protein. Binding affinities were determined, and data were analyzed as for Table 1. Affinities for rCB1 (from Table 1) are included for comparison.

**Table 3:**
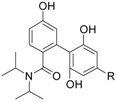
Affinities of biphenyl analogs for rCB1 and mCB2 cannabinoid receptors (± 95% confidence limits).

Compound	R	*K*_i_ (nM) ^1^		rCB1/mCB2
		rCB1	mCB2	
**23c** **AM4353**	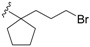	229 ± 70	10.5 ± 3.5	21.8
**23a** **AM4349**	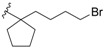	702 ± 220	22.4 ± 4.5	31.3
**23b** **AM4344**	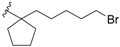	745 ± 215	17.4 ± 4.8	42.8
**23d** **AM4347**	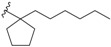	341 ± 100	5.7 ± 1.5	59.8

^1^ Cannabinoid receptors were prepared, binding affinities measured, and data analyzed as described for Table 1.

**Table 4:** Functional potencies (EC_50_) of key cannabilactones and CP-55,940 for the hCB2 cannabinoid receptor (± 95% confidence limits).

Compound	*EC*_50_ (nM)^1^ (classification)	*E(max)* (%) ^2^
	hCB2	
**CP-55,940**	3.4 ± 1.2 (agonist)	100
**1a** **AM1710**	10.5 ± 2.5 (agonist)	73
**1b** **AM1714**	36.9 ± 6.8 (agonist)	77
**3f** **AM4346**	3.7 ± 1.5 (agonist)	89

^1^ Functional potencies at hCB2 receptor were determined by measuring the decrease in forskolin-stimulated cAMP levels [38,57]. EC_50_ values were calculated using nonlinear regression analysis. Data are the average of two independent experiments run in triplicate. ^2^Forskolin stimulated cAMP levels were normalized to 100%. E(max) is the maximum inhibition of forskolin stimulated cAMP levels and is presented as the percentage of CP-55,940 response at 500 nM.
